# The influence of proteolytic enzymes on the change of lysozyme properties

**DOI:** 10.1371/journal.pone.0326386

**Published:** 2025-06-26

**Authors:** Łukasz Tomczyk, Grzegorz Leśnierowski, Aneta Tomczak, Jubilee Amajuoritse Ajemigbitse, Tomasz Szablewski, Renata Cegielska-Radziejewska

**Affiliations:** 1 Department of Food Quality and Safety Management, Poznan University of Life Sciences, Poznan, Poland; 2 Department of Food Biochemistry and Analysis, Faculty of Food Science and Nutrition, Poznan University of Life Sciences, Poznan, Poland; Harvard University Medical School, UNITED STATES OF AMERICA

## Abstract

This research examines the enzymatic modification of lysozyme, a glycosidic hydrolase that has restricted effectiveness against Gram-negative bacteria, in order to produce bioactive peptide fractions with improved antibacterial and physicochemical characteristics. Utilizing chicken egg lysozyme, modifications were performed in controlled settings with proteolytic enzymes, mainly pepsin, and experiments with a pepsin-trypsin ratio. The modification methods sought to improve the hydrophobic nature of lysozyme’s surface, create oligomeric and peptide forms, and decrease immunogenicity. Findings showed that raising pepsin concentration enhanced the creation of peptide fractions, increasing surface hydrophobicity while reducing hydrolytic and antioxidant activities. Increased hydrophobicity and reduced enzyme activity were linked to enhanced antibacterial effectiveness, particularly against Gram-negative bacteria, a characteristic absent in natural lysozyme. Additionally, the research noted a decrease in immunoreactivity as pepsin concentrations increased, achieving the lowest antibody response in optimized formulations. This enzymatic method offers an economical way to create lysozyme derivatives that hold considerable promise for wider applications, particularly in scenarios where lower immunoreactivity and a prolonged antibacterial spectrum are needed.

## Introduction

Lysozyme, obtained from the protein fraction commonly known as an enzyme derived from the group of glycosidic hydrolases, is widely used in various fields of industry and science [1]. The specificity of this protein derives from its structural structure because its active site participates in the process of decomposition of β-(1–4) glycosidic bonds located between N-acetylmuramic acid and N-acetylglucosamine in the bacterial cell wall [2]. However, due to its specific structure, the antibacterial activity of the native enzyme is limited only to Gram-positive bacteria [3,4]. A significant part of bacteria, both pathogenic and causing spoilage during storage, are Gram-negative bacteria. In order to increase the use of lysozyme also towards Gram-negative bacteria, it should be adapted accordingly, i.e., to obtain a form that will be active against this type of bacteria [5,6]. Additionally, lysozyme serves as an excellent candidate for antimicrobial peptide research due to its well-characterized structure and ease of modification. Its stability, established enzymatic function, and the ability to undergo controlled proteolytic processing make it a valuable model protein for developing novel antimicrobial agents. Currently, there are several methods known for modifying lysozyme to obtain a dimeric or peptide form in which the hydrophobic surface originally enclosed within the enzyme molecule is exposed and its active centre is opened. The increase in lysozyme surface hydrophobicity, achieved through enzymatic hydrolysis that exposes previously buried hydrophobic regions, can enhance its ability to interact with bacterial membranes, particularly those of Gram-negative bacteria. Such modifications lead to the formation of new products – bioactive lysozyme derivatives, with completely new physicochemical and antibacterial properties and, thus new application potential [7–9]. The production of biologically active peptides can take place, among others, by means of enzymatic hydrolysis. One of the most common enzymes used for enzymatic hydrolysis of stem proteins is pepsin. Its concentration in relation to lysozyme plays an important role during this process [10–12]. Beyond modifying the antibacterial spectrum of lysozyme, enzymatic hydrolysis may also influence its allergenic potential. Since protein allergenicity is often linked to intact conformational epitopes, enzymatic cleavage leading to smaller peptides and oligomeric forms could reduce immunogenicity. This is particularly relevant in food and pharmaceutical applications, where lysozyme is widely used but can pose an allergenic risk to sensitive individuals. Furthermore, in this study, we explored a broader range of enzyme-to-substrate ratios than in previous research, providing a more detailed insight into how enzymatic hydrolysis affects lysozyme’s structure and function [13].

Preliminary studies have shown that the use of hydrolytic enzymes, mainly pepsin but also trypsin, makes it possible to obtain, under strictly defined conditions, modified lysozyme biopeptide preparations showing new, valuable properties compared to native lysozyme [14]. The possibility of obtaining an attractive product with high application potential in a relatively easy and inexpensive way prompted us to continue work on this method, and the first goal of the research presented in this article was to further optimize the conditions of the modification process, this time with the participation of a mixture of proteolytic enzymes. The effect of using a mixture of pepsin and trypsin enzymes on the efficiency and quality of the modification process was studied in different ways. The second objective was to perform lysozyme modification using pepsin at different concentrations in strictly controlled conditions of the reaction environment in order to obtain the largest number of peptide fractions. The resulting formulations were evaluated for their physicochemical properties and their immunoreactivity was tested.

## Materials and methods

### Materials

The research material was a commercial preparation of lysozyme monomer in a powdered form with an activity of 21,252 U/mg, isolated from chicken egg white (Ovopol, Poland).

### Enzyme modification of lysozyme

Enzymatic modification of lysozyme was carried out in two successive steps.

1. Stage one – hydrolytic modification of lysozyme using pepsin and trypsin. For modification, a 5% (w/v) aqueous solution of lysozyme at pH 2 was prepared. The buffer for this solution was prepared by dissolving potassium dihydrogen phosphate in deionized water, and the pH was adjusted to 2.0 using concentrated hydrochloric acid, as measured with a pH meter. A mixture of trypsin (trypsin from porcine pancreas, powder, Sigma-Aldrich) and pepsin (pepsin from porcine gastric mucosa, powder, Sigma-Aldrich) in the ratios of 1/0, 0.75/0.25, 0.5/0.5, 0.25/0.5, and 0/1, respectively. The hydrolysis reaction was carried out in the ratio of lysozyme to the enzyme mixture of 500/1 (w/w). The reference sample was a lysozyme solution without added enzymes. Samples containing pepsin and trypsin and the reference sample were modified in a BUCHI Syncore® analytical reactor (Switzerland) at 55 °C for 60 minutes, after which the reaction was stopped by heating them for 5 minutes at 85 °C and then, after cooling, their pH value was set at 7.0.
2. Stage two – consisted of assessing the impact of the peptides and oligomers created as a result of the lysozyme modification on the physicochemical properties. For this purpose, further lysozyme modifications were performed this time on the lysozyme to pepsin ratio: 1/2000, 1/1500, 1/750, 1/500, 1/250, 1/166, 1/125, 1/100 (w/w) for the most preferred embodiment of the experiment in the formation of peptide and oligomeric fractions of the enzyme, i.e., pepsin.

The adopted enzyme ratio was based on the author’s previous experience [13], which analyzed the impact of different proteolytic enzyme ratios on model protein substrates.

The resulting formulations were frozen and then lyophilized in a freeze-dryer (Labconco, Kansas City, MO, USA). Preparations prepared in this way were subjected to analytical tests specified in section.

### Analytical procedures

#### Electrophoresis.

The electrophoretic analysis was conducted in accordance with the method described by Leśnierowski [15]. The samples (applied in a volume of 1.5 µL) were separated by electrophoresis on gradient gels 16.5% MP Tris-Tricine (4563064, Bio-Rad, Hercules, CA, USA) in a volume of 5 µL. Samples obtained after the modification were dissolved in sample buffer (30% glycerol, 0.3% TRIS-HCl (pH 6.8), 6% SDS, 0.1% bromophenol blue) and heated at 100 °C for 5 minutes. 3 µL of the samples was applied onto the stacking gel. The current intensity during electrophoretic separation was 60 mA and 90 mA for the concentrating and separating gels, respectively. After electrophoresis, the gel was fixed for 1 hour in a solution consisting of 40% water, 50% methanol, and 10% acetic acid. The gel was then stained for 20 hours in 10% acetic acid with the addition of 0.25 g of Coomassie brilliant blue R-250 for 20 h. Then, the gels were decolorized in 10% of an aqueous solution of acetic acid until their background was completely discoloured. The decolorized gels were scanned and their images were stored as computer files. The quantitative content of individual fractions of lysozyme in the preparations obtained after the modification was determined densitometrically using the TotalLab Quant software 1.5.170 (Nonlinear Dynamics Ltd., Gosforth, UK)

#### Capillary electrophoresis profile.

The capillary electrophoresis method was used to determine the molecular weights of the obtained peptide or oligomeric fractions. This analysis is similar to high-performance liquid chromatography, but it is more sensitive and can separate small molecules that classical HPLC cannot always resolve. The system provides automatic and rapid separation of protein fractions facilitating the characterization of proteins and peptides. It is an excellent alternative and a more sensitive method of protein fraction analysis. Proteins and peptides were subjected to molecular analysis using a commercial assay for the analysis of protein fractions and peptides up to 250 kDa of the highly sensitive Agilent High Sensitivity Protein 250 Kit (5067−1575, Agilent Technologies, USA) at a sensitivity of 1 μg/μL. Samples were prepared according to the description included with the kit – Agilent High Sensitivity Protein 250 Kit. The results were processed using Agilent 2100 Expert Software (Agilent Technologies, USA) as described by Herwig et al. [16].

#### Western-blot method.

Protein fractions, after previous separation by SDS-PAGE electrophoresis, were transferred to a polyvinylidene difluoride membrane with a special porosity of 0.2 μm (Immobilon-P, Merck Millipore Ltd.). Capillary transfer was used according to the method described by Zeng et al. [17].

Sera of 5 outpatients diagnosed with egg allergy were obtained from the Allergy Diagnostics and Treatment Center SNOZ Alergologia Plus in Poznań (Poland). The Bioethics Committee at the Poznań University of Medical Sciences accepted the application for the use of biological material (agreement no. 671/17 of 2017 and annex 516/19), which guarantees compliance with the required ethical standards. The obtained characteristics of the sera are presented in Table 1. The ELISA method (Polycheck, Germany) was used to determine the antibody class.

**Table 1:** Characterization of the physicochemical properties of the lysozyme preparations obtained with different addition of a mixture of proteolytic enzymes.

No.	Trypsin to Pepsin Ratio	Amount of Peptide Fraction (%) (Mean ± SD)	Amount of Oligomers Fraction (%) (Mean ± SD)	Hydrolytic Activity (U/µL) (Mean ± SD)	Change in Hydrophobicity (%) (Mean ± SD)	Antioxidant Properties (TE/mgL) (Mean ± SD)
1	0/0	0a ± 0	0a ± 0	21191.52f ± 131.17	7.06a ± 0.05	74.68a ± 0.61
2	1.00/0.00	0a ± 0	0a ± 0	19442.20e ± 161.75	7.54a ± 0.08	147.40e ± 1.19
3	0.75/0.25	19.38b ± 0.33	0a ± 0	10692.70d ± 178.04	11.19c ± 0.11	107.64d ± 0.76
4	0.50/0.50	28.22c ± 0.57	0a ± 0	7581.92c ± 142.11	12.99d ± 0.12	91.76c ± 0.62
5	0.25/0.50	38.23d ± 0.58	2.10b ± 0.03	6610.34b ± 110.09	13.44e ± 0.11	76.46a ± 0.49
6	0.00/1.00	46.54e ± 0.43	3.20b ± 0.03	4595.20a ± 75.26	21.98f ± 0.36	69.66b ± 0.42

Values are presented as mean ± standard deviation (SD). Different superscript letters indicate statistically significant differences between groups (p ≤ 0.05, Tukey’s test).

Sera were diluted in 1% BSA in TBS-Tween (1:20 v/v). Monoclonal antibodies recognizing IgE labeled with human alkaline phosphatase (A3076, Sigma Aldrich, USA) were used as Ab II antibodies (17). Membranes were subjected to array analysis using the CLIQS program (TotalLab Quant, Great Britain).

#### Hydrolytic activity.

The hydrolytic activity of obtained preparations was determined by spectrophotometric method described by Leśnierowski [15]. The method uses the phenomenon of reducing the turbidity of the bacterial suspension of *Micrococcus lysodeikticus* (Sigma-Aldrich Co., USA) as a result of adding a sample to it. Absorbance was measured at a wavelength of λ = 450 nm at a temperature of 21 °C. The analysis was conducted with a VSU2-P Carl Zeiss Jena spectrophotometer (VSU2-P, Oberkochen, Germany).

#### Hydrophobicity of modified lysozyme.

The surface hydrophobicity of the sample was determined using the ligand agent polyoxyethylene sodium (Tween 80, SERVA, Germany). The method was developed by Lieske and Konrad [18] and modified by Leśnierowski [15]. This detergent has the property of preventing the Bio-Rad dye from binding to the modified lysozyme sample molecule by coating the protein with a hydrophobic site, which causes binding to the hydrophobic sites of the protein and the dye complex (measurable).

#### Antioxidative activity.

The antioxidant activity of the preparations was analyzed using the ABTS (2,2’-azino-bis(3-ethylbenzothiazoline-6-sulfonic acid)) radical cation decolorization assay, as described by Re et al. [19]. This method is based on the ability of antioxidants to neutralize the ABTS• ⁺ radical cation, which results in a reduction of its characteristic blue-green color. To generate the ABTS• ⁺ radical, ABTS was reacted with potassium persulfate and incubated in the dark at room temperature for 12–16 hours before use. The radical solution was then diluted with ethanol or phosphate-buffered saline (pH 7.4) to obtain an absorbance of approximately 0.70 (± 0.02) at 734 nm. The antioxidant activity of the samples was determined by mixing a defined volume of the sample with the prepared ABTS• ⁺ solution, followed by measuring the decrease in absorbance at 734 nm after a specific incubation time (typically 6–10 minutes). The results were expressed as millimolar Trolox equivalent per milliliter of sample (mM TE/mL), allowing for a standardized comparison of antioxidant capacities relative to the reference antioxidant, Trolox (Sigma-Aldrich, Munich, Germany).

#### Microbiological test.

The antibacterial activity of the monomer and modified lysozyme preparations against Gram-negative bacterial strains (*Escherichia coli* PCM 2793, *Proteus mirabilis* PCM 1361, *Salmonella enteritidis* PCM 941, *Pseudomonas fluorescens* PCM 3107, *Pseudomonas fragi* PCM 1856) (Hirszfeld Institute of Immunology and Experimental Therapy Wrocław, Poland) was assessed. Bacterial suspensions were prepared in 0.85% NaCl (Biomérieux, Marcy-l’Étoile, France) with a density of 0.5 (McFarland scale) in a Densimat apparatus (Biomérieux) for 24 hours. Decimal dilutions were prepared successively: for *Listeria innocua* bacteria — 10^7^ CFU/mL, for other bacterial strains — 10^5^ CFU/mL. At the same time, aqueous solutions of lysozyme were prepared at a concentration of 1%. The optical density of lysozyme and bacteria was measured using Bioscreen C (Lab systems Helsinki, Finland). Optical density was measured at 420–580 nm. Samples analyzed in the experiment were made from 30 µL of bacterial inoculum, 150 µL of lysozyme solution, and 120 µL of nutrient broth. Then, the samples were incubated at 37 °C for 72 hours. Changes in the optical density of the sample during the experiment were recorded automatically every 30 minutes for 96 hours.

### Statistical analysis

All results were collected and analysed using Statistica 13.2 (Tibco Software, Palo Alto, CA, USA), and the results are presented as the mean values with the standard error. Analysis of variance and post hoc analysis with the Tukey test were used to verify results with heterogeneity of variance. Each test was performed in quintuplicate.

## Results

The aim of the research was to obtain bioactive peptide fractions from lysozyme derived from chicken eggs by enzymatic hydrolysis. The results presented in this article are a continuation of research on a new method of lysozyme modification carried out in the environment of hydrolytic enzymes. Past studies have shown that using hydrolytic enzymes, especially pepsin but also trypsin, in the process of lysozyme modification under strict conditions resulted in obtaining biopeptide preparations with new and valuable properties compared to native lysozyme. Taking into account the results obtained in the preliminary studies, the modification process was carried out at a constant pH of the reaction mixture equal to 2, a temperature of 55 °C for a period of 60 minutes, using a wider range of enzyme concentrations [14].

**Fig 1:**
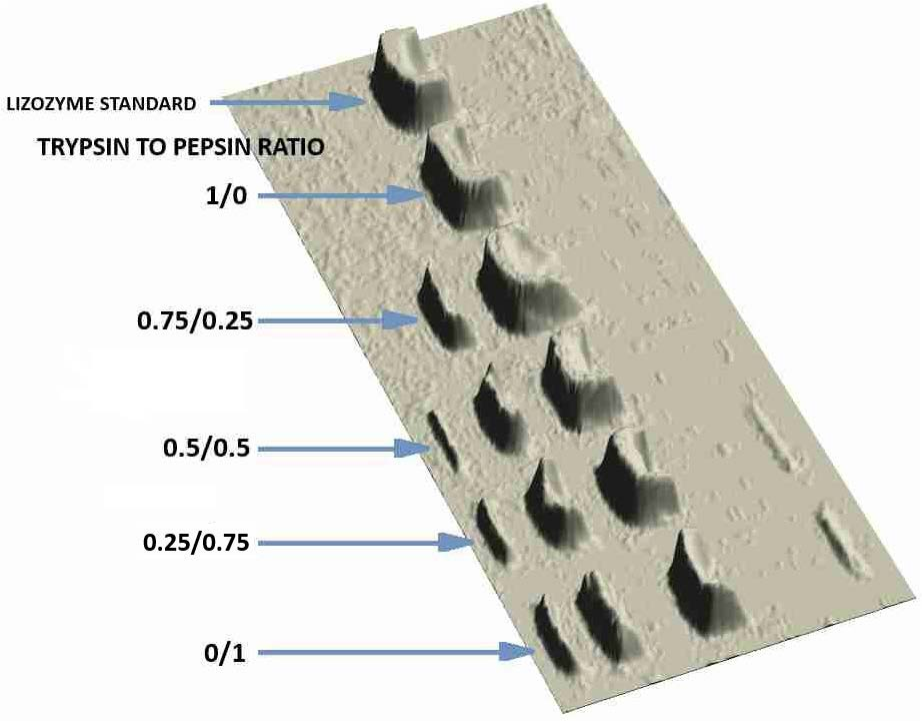
Electrophoretic image of modified lysozyme preparations with different amounts of pepsin and trypsin enzyme mixtures.

The modifications carried out in the first stage of the study showed that the use of the enzyme mixture produced interesting results. The test results obtained are shown in Table 1, while Fig 1 shows the results of electrophoretic analysis in the form of a 3D image illustrating the formation of a new form of lysozyme depending on the conditions used for its modification.

Preparations obtained by lysozyme modification using a mixture of proteolytic enzymes at various concentrations contained, in addition to the monomer, peptide, and oligomeric forms (Table 1). It was shown that the direction of changes in physicochemical properties was analogous in comparison to other modifications carried out with the use of hydrolytic enzymes. However, it was found that higher pepsin concentrations enabled a more efficient formation of oligomeric and peptide forms. Only in the case of samples containing lysozyme without the participation of proteolytic enzymes were no peptide and oligomeric fractions demonstrated. In the case of samples where the trypsin/pepsin ratio of the modification was 0.75/0.25, the formation of 1 peptide fraction was evidenced. The increase in pepsin concentration in the enzyme mixture resulted in the appearance of 2 peptide fractions. In fact, the greatest amount of peptide forms was obtained when pepsin alone was used as a modifying agent (F (5;24) =12672; p < 0.001). The oligomerization process was obtained in the case of samples using a mixture of trypsin and pepsin at ratios of 0.25/0.75 and 0/1.

Analysing the subsequent results of the modifications performed, it was found that significant changes in the surface hydrophobicity (F(5;24)=235974; p < 0.001) of the modified lysozyme, as well as its hydrolytic activity (F(5;24)=208385.2; p < 0.001) and antioxidant activity (F(5;24)=250417.8; p < 0.001), were significantly dependent on the concentrations of proteolytic enzymes. Based on the results of statistical analysis, it was shown that the factor determining the effectiveness of the hydrolysis process was pepsin concentration. It’s a higher share in the mixture of hydrolytic enzymes guaranteed obtaining the preparations with the greatest increase in surface hydrophobicity. The use of the highest concentration of pepsin in the modification process of lysozyme increased its surface hydrophobicity by 14.83%. There were no significant differences in surface hydrophobicity between the sample without hydrolytic enzymes and the sample with the highest trypsin concentration. Therefore, it can be concluded that the participation of trypsin in the enzyme mixture has a much weaker effect than pepsin on the structural changes of the lysozyme molecule responsible for moving its hydrophobic interior to the enzyme surface. The opposite effect was achieved in the case of antioxidant activity, where the use of trypsin was more effective than the use of pepsin. The influence of the conducted lysozyme modification processes on its hydrolytic activity was also demonstrated. This was different from the hydrophobic changes because the increase in pepsin concentration significantly reduced lysozyme activity. It was shown that the higher the pepsin concentration, the lower the hydrolytic activity obtained (Table 1). It should be emphasized that all the lysozyme modifications used so far by the authors, especially thermal ones, showed a reduction in the hydrolytic activity of the enzyme compared to its native form. However, this did not adversely affect the antibacterial properties of the lysozyme; on the contrary, the enzyme activity against bacteria was often more effective than the native enzyme, and in addition, the modified lysozyme was characterized by effective action against Gram-negative bacteria.

The results of previous studies and the results obtained in the first stage indicate the influence of pepsin concentration on the lysozyme modification effect, contributed to conducting studies in which the spectrum of pepsin concentrations was extended [14]. The researchers adopted a study system that used the most favourable conditions of native pepsin lysozyme modification from the first part of the work. Modifications of the lysozyme were made according to the procedure given in the material and methods section.

**Fig 2:**
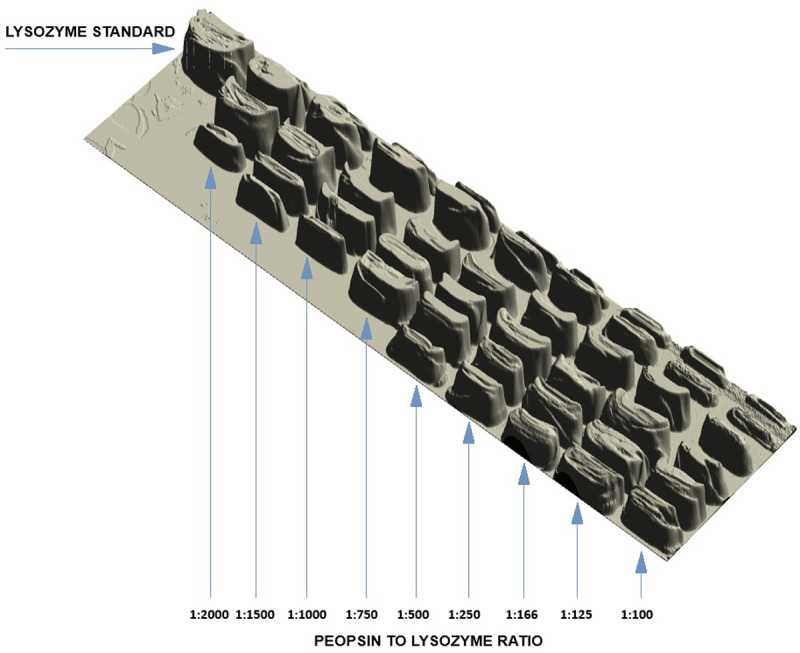
Electrophoretic image of modified lysozyme preparations with different amounts of pepsin.

Analogously, as in the first stage of the research, the modified lysozyme preparations were obtained, containing both the monomer as well as the peptide and oligomeric fractions. The results of analytical tests of preparations after modification of native lysozyme with different concentrations of pepsin are presented in Table 2, and the results of electrophoretic analysis are presented in Fig 2. In addition, capillary electrophoresis was also performed to determine the molecular weights of the resulting peptide and oligomeric fractions. In the final stage of the research, the effect of modification conditions with the participation of pepsin on the allergic immunoreactivity of the obtained lysozyme preparations was evaluated.

**Table 2:** Characterization of physicochemical properties of obtained lysozyme preparations with different lysozyme to pepsin ratios.

No.	Pepsin to Lysozyme Ratio	Peptide Fraction (%)	Oligomers Fraction (%)	Hydrolytic Activity (U/µL)	Change in Hydrophobicity (%)	Antioxidant Properties (TE/mgL)
7	0/1	0a ± 0	0a ± 0	21128.11j ± 157.03	0a ± 0	74.96h ± 0.67
8	1/2000	17.01b ± 0.09	23.18j ± 0.14	9093.04i ± 80.579	7.36a ± 0.07	73.04h ± 1.14
9	1/1500	29.35c ± 0.07	22.37i ± 0.14	8172.91h ± 506.84	9.65b ± 0.10	72.56g ± 0.66
10	1/1000	37.20d ± 0.10	19.34h ± 0.09	7013.52g ± 335.45	12.69c ± 0.12	71.57f ± 0.62
11	1/750	45.02e ± 0.09	14.32g ± 0.10	5881.37f ± 237.46	16.43d ± 0.20	71.40f ± 0.41
12	1/500	64.04f ± 0.10	12.32f ± 0.10	4487.22e ± 26.40	21.87fe ± 0.31	69.02d ± 0.56
13	1/250	72.16g ± 0.15	9.29e ± 0.02	4294.08d ± 28.86	29.77f ± 0.19	67.62c ± 0.93
14	1/166	82.82h ± 0.14	7.76d ± 0.01	4041.76c ± 29.42	32.54g ± 0.26	65.47b ± 0.30
15	1/125	85.61i ± 0.14	4.60c ± 0.01	3874.24b ± 19.59	41.14h ± 0.41	63.07a ± 0.68
16	1/100	85.19i ± 0.50	2.31b ± 0.01	3565.52a ± 20.06	53.79i ± 0.26	62.73a ± 0.57

Values are presented as mean ± standard deviation (SD). Different superscript letters indicate statistically significant differences between groups (p ≤ 0.05, Tukey’s test).

As a result of enzymatic modification of lysozyme using pepsin, regardless of its concentration, two fractions were obtained – monomeric and peptide. The number of individual peptide fractions was dependent on the pepsin concentration. Two peptide fractions with an average molecular weight of 11,82 kDa and 10,18 kDa were obtained in a 1/2000 ratio for pepsin to lysozyme-modified samples. In turn, further increasing the amount of pepsin relative to lysozyme contributes to the formation of the third peptide fractions with a molecular weight of 7,53 kDa. A fourth peptide fraction with an average weight of 6,47 kDa was determined for samples using a pepsin to lysozyme ratio of 1/500–1/100 (Table 3). Analysing the obtained results, it was found that the amount of peptide fraction as a result of lysozyme modification is significantly dependent on pepsin concentration (F (9;162) =41863; p < 0.001). It was also shown that the increase in pepsin concentration significantly affected the efficiency of oligomerization. As the concentration of pepsin increases during the modification, the number of oligomeric forms decreases (F (9;162) =2871; p < 0.001). The highest percentage of peptides, 85.5%, was obtained in the case of using a pepsin to lysozyme ratio of 1/125, while a further increase in the pepsin ratio at the time of modification did not significantly affect the greater proportion of peptide forms. The lowest percentage of peptides, 17.08%, was obtained for the variant of the study in which the percentage of pepsin to lysozyme was the lowest (1/2000). The opposite relationship was found in the case of determining the amount of oligomeric fractions in lysozyme preparations after modification. The ratio of pepsin to lysozyme, 1/2000, during modification contributed to the highest amount of lysozyme. 23.31% oligomeric forms (Table 2) with an average molecular weight of 24.89 kDa were obtained (Table 3).

**Table 3:** Molecular masses of the fractions obtained as a result of lysozyme modification with pepsin.

Fraction	Size [kDa]
	Mean ± SD
Oligomeric	24,89 ± 2,76
Lysozyme	14,12 ± 0,346
Peptide 1	11,82 ± 0,457
Peptide 2	10,18 ± 0,315
Peptide 3	7,53 ± 0,332
Peptide 4	6,47 ± 0,0186

The conducted studies also showed the significance of the influence of pepsin concentration during the modification process on changes in enzyme hydrophobicity. It was found that the highest mean increase in surface hydrophobicity of 53.71% occurred in the pepsin-to-lysozyme modified sample 1/2000, and the lowest (7.36%) was observed in the 1/100 modified sample. It can be concluded that the number of released peptides and their content in a given preparation were of decisive importance for the increase in surface hydrophobicity. The more peptides were measured in the preparation, the higher the surface hydrophobicity. The release of peptides resulted in the exposure of the hydrophobic interior of lysozyme to its surface.

The modification conditions applied in the second stage of the experiment also significantly affected the obtained values of the other tested physicochemical parameters, i.e., hydrolytic activity (F (9;162) =5641; p < 0.001) and antioxidant activity (F (9;162) =64173; p < 0.001) of the modified lysozyme. The highest antioxidant activity of 73,31 TE/mgL on average, did not differ statistically significantly from the activity of native lysozyme and was characterized by the preparations with the smallest share of pepsin in the reaction mixture. Further increases in pepsin in the lysozyme modification process resulted in a decrease in its antioxidant activity. A similar relationship was found when evaluating the hydrolytic activity of lysozyme preparations after modification. The highest value of hydrolytic activity was achieved using the least drastic modification conditions, i.e., the lowest pepsin concentration (Tables 2 and 4) .

**Table 4:** Presentation of used sera.

Serum	Allergy to			
	Egg white		Egg yolk	
	Class	IgE concentration (kU/L)	Class	IgE concentration (kU/L)
I	3	3.5–17.5	3	3.5–17.5
II	4	~50	3	3.5–17.5
III	6	>100	3	3.5–17.5
IV	4	~50	4	~50
V	3	3.5–17.5	3	3.5–17.5

### Western-blot and array analysis

**Fig 3:**
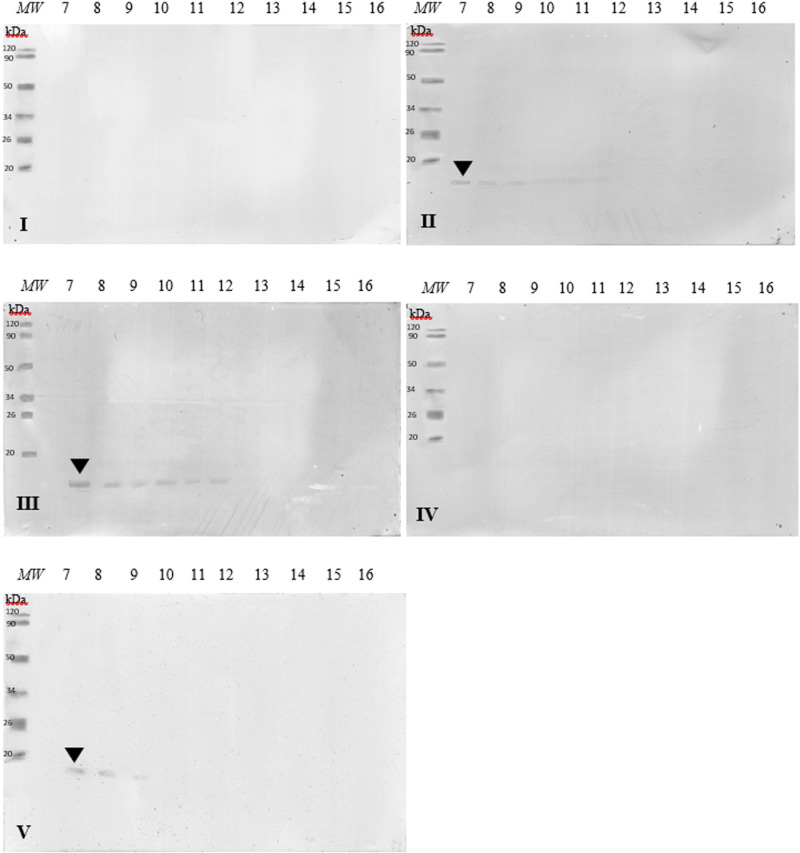
Images of the membrane obtained as a result of the Western-blot analysis with the sera of five patients allergic to eggs (I–V) for lysozyme extracts. MW – molecular weight marker, 7-16 lysozyme samples, black arrowhead – fraction ~15 kDa.

**Fig 4:**
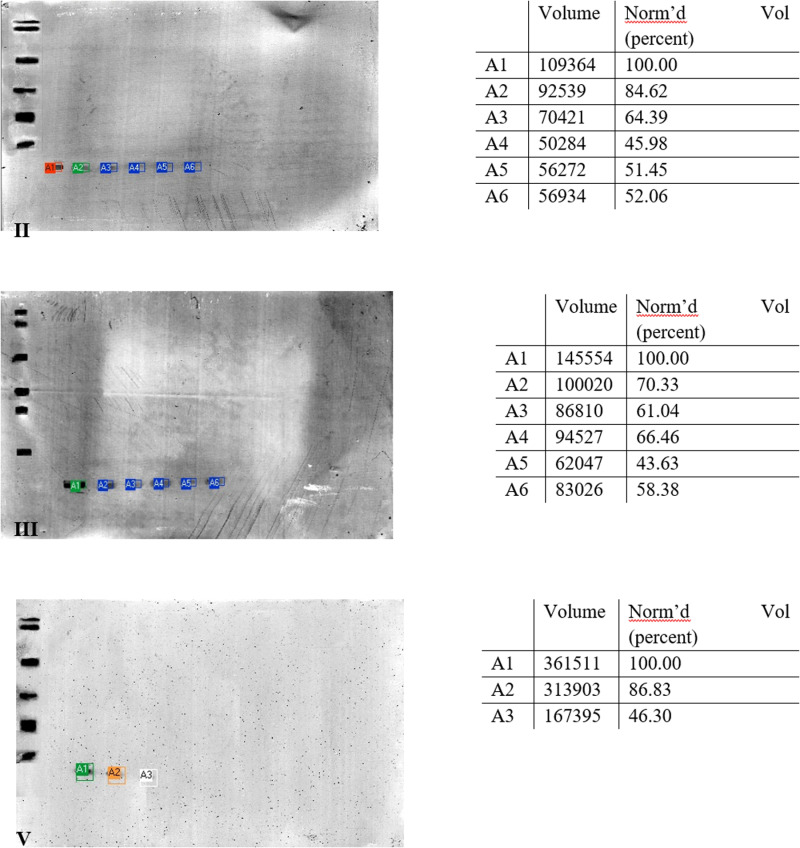
Images of the membrane obtained as a result of the Western-blot analysis after optical density analysis.

The images of membranes after western blot analysis, where antibodies contained in the patients’ sera recognized allergenic epitopes in the tested lysozyme samples, were subjected to array analysis (Fig 4). This analysis aims to determine the optical density of a given protein detected by antibodies through the colour intensity. In 3 of 5 sera used, antibody reactions with lysozyme fractions were observed (Fig 3). When the optical density is lower, the immunoreactivity of the allergenic fraction of chicken egg white, i.e., lysozyme, decreases. Pure lysozyme (A1) was chosen as 100% optical density.

In the case of sera number II and III, the antibodies contained therein recognized allergenic epitopes in the 6 lysozyme samples tested. Antibodies contained in serum no. V recognized protein fractions in the first 3 attempts (1–3). The antibodies in each reaction recognized only the approximately 15 kDa protein (black arrowhead, Fig 3). The strength of the reactions varied.

Allergen Nomenclature WHO/IUIS Allergen Nomenclature Sub-Committee currently recognizes 10 allergenic fractions of hen eggs. Gal d 4 is a lysozyme with a molecular weight of ~14.3 kDa. Western blot analysis using serum from children diagnosed with allergy to hen egg protein fractions showed that 3 out of 5 patients were allergic to the Gal d 4 fraction – serum II, III, and V (Fig 3). The antibodies recognized epitopes of the protein fraction of about 15 kDa. In the case of samples II and III, the antibodies recognized samples numbered 7–12, where sample no. 7 was pure lysozyme not subjected to digestion. Antibodies contained in serum V recognized epitopes in samples numbered 7–9 (Fig 3). Of the five patients studied, 2 did not show any reaction to the tests, which confirms the allergic reactions as individual responses, and it can be assumed that these patients were not allergic to Gal d 4 but to other egg-allergenic determinants. Studies show that with progressive digestion of lysozyme, the antibody reaction was weaker and after the 13th attempt it disappeared completely. Therefore, it can be assumed that the digestion of lysozyme samples resulted in a reduction and subsequent cessation of the allergenic immunoreactivity of the Gal d 4 fraction. Membrane images subjected to optical density testing showed a decrease in the intensity of the antibody reaction based on the intensity of the burst by initially 25% to about 50% (Fig 4). In summary, the studies demonstrate a reduction in the content of the allergenic determinant of lysozyme and thus its immunoreactivity. Research should be expanded to include the number of patients.

The obtained results of studies of changes in the physicochemical properties of lysozyme after the modification process using the enzymes trypsin and pepsin are an indication to conduct tests on the antibacterial activity of the obtained lysozyme preparations. Full studies will be presented in a separate paper. So far, preliminary tests have been carried out to assess the effect of native lysozyme, as well as the highest concentrations of trypsin- and pepsin-modified lysozyme preparations obtained in Part I of the study, on selected Gram-negative bacteria (Table 5). It has been shown that the inhibition of growth of the tested bacterial strains was achieved only with the lysozyme preparation obtained by pepsin modification – Trypsin to pepsin ratio 0.00/1.00. In contrast, the lysozyme monomer and the enzyme preparation after trypsin modification (Trypsin to pepsin ratio 1.00/0.00) were not found to be effective against the tested Gram-negative bacterial strains (Table 5). This result indicates that the antibacterial activity of lysozyme could be expanded as a result of its modification with pepsin. Therefore, the antibacterial activity of the modified lysozyme preparations obtained in the stage II of the study is currently being evaluated.

**Table 5:** Microbiological test of lysozyme activity against selected Gram-negative bacteria.

Gram (-) bacteria	Concentration 1%		
	Native lysozyme	Trypsin to pepsin ratio 1.00/0.00	Trypsin to pepsin ratio 0.00/1.00
*Salmonella enteritidis*	–	–	+
*Proteus mirabilis*	–	–	+
*Escherichia coli*	–	–	+
*Pseudomonas fluorescens*	–	–	+
*Pseudomonas fragi*	–	–	+

− no effect on bacteria, + inhibition of bacterial growth

## Discussion

The authors presented earlier research results on the enzymatic method of lysozyme modification, indicating that the hypothesis regarding the possibility of using proteolytic enzymes to modify the enzyme is not only an interesting idea but also an exceptionally attractive and effective technology for producing its modified form [14]. The research results presented in this paper also showed the possibility of obtaining preparations with a high degree of hydrolysis and a significantly increased hydrophobic surface. In the first part of the experiment, the most beneficial hydrolytic effect was obtained using the highest addition of pepsin in the reaction mixture, while with the increase in the addition of trypsin, a lower degree of lysozyme hydrolysis was obtained. This is consistent with the research of other authors, which indicates that trypsin works most effectively in combination with other enzymes, e.g., papain. The use of such hydrolytic systems usually resulted in significant, and sometimes even complete hydrolysis of lysozyme, releasing its bioactive peptides [20–22]. The addition of trypsin significantly influenced the antioxidant activity, which was significantly higher than that of the native enzyme. Studies by other authors also showed that biopeptides obtained from lysozyme were characterized by increased antioxidant activity [21,23,24]. Another research showed that enzymatically hydrolyzed lysozyme from hen egg white can release antimicrobial peptides, further enhancing its bioactivity [25].

In the second part of the experiment, as a result of modification of lysozyme using only pepsin, under the most favourable conditions, preparations containing dimers and four peptide fractions in the amount of over 87% were obtained, the surface hydrophobicity increased by over 53%, which indicates their high quality. Changes in the hydrophobicity of enzymatically modified lysozyme clearly show that hydrolysis of the enzyme, similar to other modifications of lysozyme [1,7,26], promoted an increase in its surface hydrophobicity. Our results demonstrated that pepsin had a stronger impact on the structural changes of lysozyme compared to other proteolytic enzymes, particularly in terms of increased hydrophobicity. This effect is likely related to the specific cleavage pattern of pepsin, which preferentially hydrolyses peptide bonds near aromatic amino acids such as phenylalanine, tyrosine, and tryptophan [27,28]. Cleavage at these sites may lead to the exposure of hydrophobic regions that are typically buried within the native lysozyme structure. Consequently, this structural alteration results in increased surface hydrophobicity, which may also influence the protein’s aggregation behaviour and other physicochemical properties. Previous studies have shown that a decrease in the hydrolytic activity of the enzyme occurs when the surface hydrophobicity increases [7] and this always results in an increase in the total antibacterial activity of lysozyme. Also, the preliminary microbiological tests of modified lysozyme preparations obtained in the first stage of the study indicate that lysozyme modified with pepsin, with higher surface hydrophobicity, peptide content, and fraction of oligomeric forms, has the ability to inhibit the growth of Gram-negative bacteria. Such antibacterial activity was not found in the case of native lysozyme and lysozyme modified with trypsin. It is well known that lysozyme’s antibacterial activity against Gram-positive bacteria is partially driven by electrostatic interactions between its positively charged residues and the negatively charged components of the bacterial cell wall, such as teichoic acids. In contrast, Gram-negative bacteria possess an outer membrane primarily composed of lipopolysaccharides and phospholipids, which act as a barrier to native lysozyme. When modifying lysozyme to increase its surface hydrophobicity, a shift in its antibacterial mechanism can be observed. While the reduction in electrostatic interactions might limit its effectiveness against Gram-positive bacteria, the enhanced hydrophobicity facilitates stronger interactions with the lipid-rich outer membranes of Gram-negative bacteria, leading to membrane destabilization and bacterial inhibition. This suggests that modified lysozyme variants, despite a decrease in hydrolytic activity, may rely on alternative mechanisms of action, such as membrane disruption, to exert their antibacterial effects. These findings align with previous studies demonstrating that increasing the hydrophobic character of antimicrobial proteins can improve their activity against Gram-negative bacteria. The antibacterial activity of lysozyme preparations modified with hydrolytic enzymes was demonstrated in the authors’ earlier studies [7,14]. The lag phase of bacteria was found to be prolonged during their incubation with lysozyme preparations obtained by modification at 70°C under various pH conditions and modification times [14].

An important consideration in lysozyme modification is its potential allergenicity. The enzymatic hydrolysis applied in this study resulted in peptide and oligomeric forms, which may reduce immunogenic potential by disrupting conformational epitopes responsible for allergic reactions. A key finding of this study was that enzymatic modification of lysozyme with pepsin led to a reduction, and eventually the complete loss, of immunoreactivity of the hydrolysed lysozyme products as the pepsin concentration in the enzyme-to-lysozyme system increased. While further studies are needed to confirm this effect, these findings suggest that enzymatically modified lysozyme could have broader applications in food and pharmaceutical formulations where allergenicity is a concern. Additionally, by evaluating lysozyme modifications across a wider range of enzyme concentrations, this study provides a more comprehensive understanding of the structural and functional changes induced by enzymatic hydrolysis, supporting the development of tailored lysozyme derivatives for specific applications.

## Conclusion

The results of this study indicate that both the use of a mixture of pepsin and trypsin enzymes and pepsin itself can induce the formation of peptide fractions and oligomeric forms of the formation of peptide and oligomeric fractions. By optimizing the conditions of modification, the greatest amount of peptide fractions are obtained from the modification, in which the hydrophobicity of the lysozyme molecule increases and its hydrolytic activity decreases. An interesting finding was the decrease in the immunoreactivity of lysozyme products observed after modification with the increase in pepsin concentration. The lysozyme modification product using a 1:250 pepsin/lysozyme ratio was completely devoid of immunoreactivity. In this case, no antibody response was demonstrated, the presence of which is a negative characteristic of native lysozyme. It can therefore be concluded that the use of proteolytic enzymes to modify the lysozyme opens up opportunities for altering its properties and for the formation of peptide fractions and requires further investigation. Therefore, it is also necessary to investigate the new activity of the modified lysozyme, especially its effect on Gram-negative bacteria. Such studies are already being carried out by our group and will be reported after their completion. Additionally, the enzymatic modification approach presents a simple, cost-effective alternative to genetic engineering techniques, making it a viable method for producing novel antimicrobial peptides.

## Supporting information

S1 Fig Raw gel image corresponding to 3D densitometric analysis shown in Fig 1. (JPG)

S2 Fig Raw gel image corresponding to 3D densitometric analysis shown in Fig 2. (JPG)

S1 Table Raw data used to generate Table 1. (XLSX)

S2 Table Raw data used to generate Table 2. (XLSX)

S1 File Capillary electrophoresis output file (.xac) from Agilent Bioanalyzer system. Contains raw signal data and electropherogram traces for samples analyzed in Table 3.
(XAC)
