# Supplementary figures and images for: The influence of proteolytic enzymes on the change of lysozyme properties
Source: PLoS One. 2025 Jun 26;20(6):e0326386. doi: 10.1371/journal.pone.0326386 (PMC12200872; doi:10.1371/journal.pone.0326386)

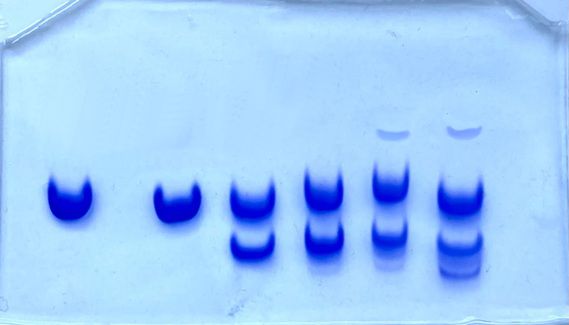

Supplement: S1 Fig — (JPG) [file pone.0326386.s001.jpg]

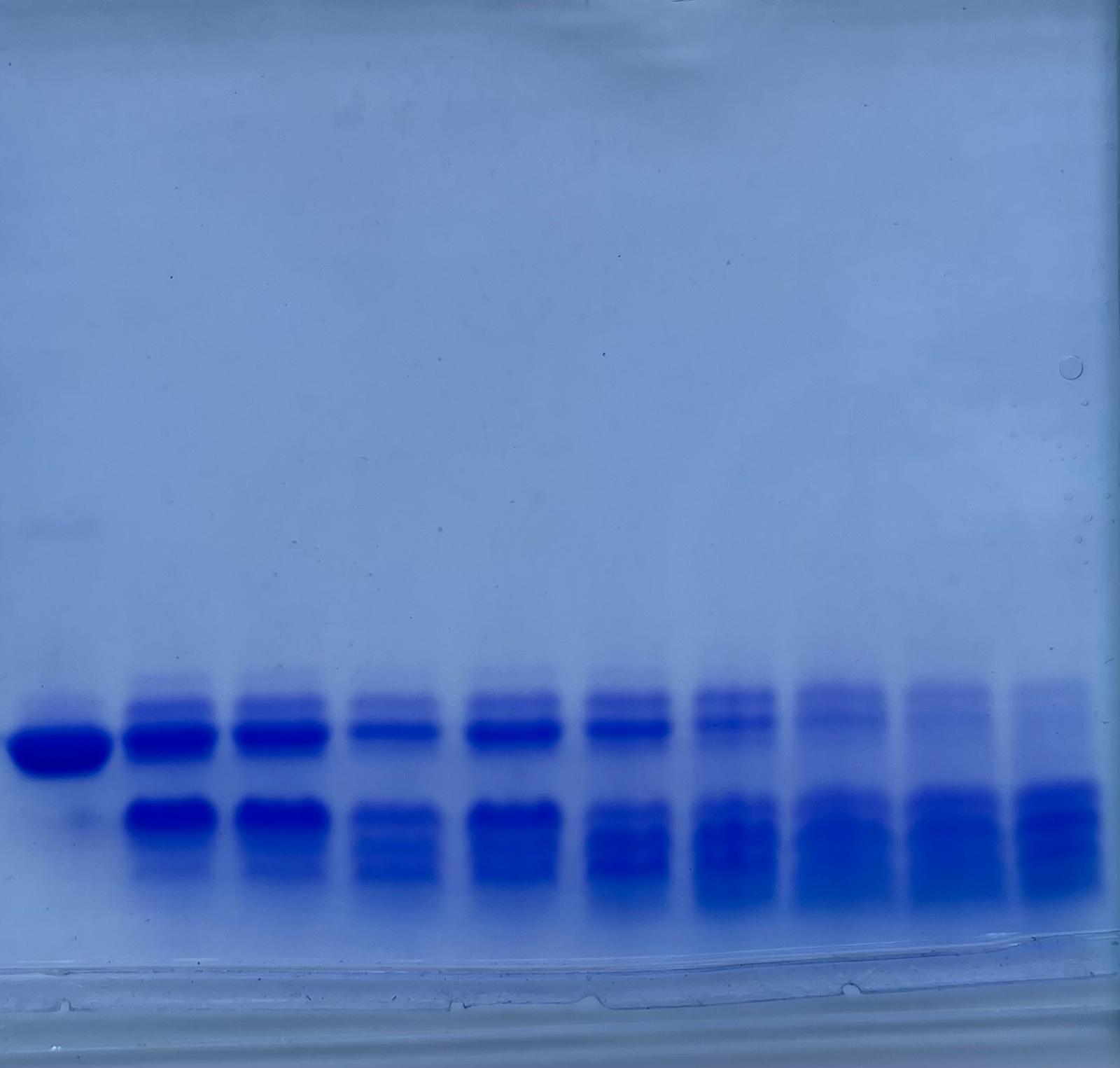

Supplement: S2 Fig — (JPG) [file pone.0326386.s002.jpg]
